# Adsorptive Removal of Copper (II) Ions from Aqueous Solution Using a Magnetite Nano-Adsorbent from Mill Scale Waste: Synthesis, Characterization, Adsorption and Kinetic Modelling Studies

**DOI:** 10.1186/s11671-021-03622-y

**Published:** 2021-11-27

**Authors:** Syazana Sulaiman, Raba’ah Syahidah Azis, Ismayadi Ismail, Hasfalina Che Man, Khairul Faezah Muhammad Yusof, Muhammad Umar Abba, Kamil Kayode Katibi

**Affiliations:** 1 Material Synthesis and Characterization Laboratory, Institute of Nanoscience and Nanotechnology (ION2), Universiti Putra Malaysia (UPM), 43400 Serdang, Selangor, Malaysia; 2 Department of Physics, Faculty of Science, UPM, 43400 Serdang, Selangor, Malaysia; 3 Department of Biological and Agricultural Engineering, Faculty of Engineering, UPM, 43400 Serdang, Selangor, Malaysia; 4 Department of Process and Food Engineering Faculty of Engineering, Universiti Putra Malaysia, 43400 Selangor, Malaysia; 5 Department of Agricultural and Bioenvironmental Engineering, Federal Polytechnic Mubi, Mubi, 650221 Nigeria; 6 Department of Agricultural and Biological Engineering, Faculty of Engineering and Technology, Kwara State University, Malete, 23431 Nigeria

**Keywords:** Magnetite nano-adsorbents (MNA), Mill scale waste, Adsorption, Copper (II) ion, Kinetic study, Adsorption isotherm

## Abstract

In this study, magnetite nano-adsorbent (MNA) was extracted from mill scale waste products, synthesized and applied to eliminate Cu^2+^ from an aqueous solution. Mill scale waste product was ground using conventional milling and impacted using high-energy ball milling (HEBM) for varying 3, 5, and 7 milling hours. In this regard, the prepared MNA was investigated using X-ray diffraction (XRD), high-resolution transmission electron microscope (HRTEM), field emission scanning electron microscopy–energy-dispersive X-ray spectroscopy (FESEM-EDS), UV–Vis spectroscopy, Fourier-transform infrared (FTIR), Brunauer–Emmett–Teller (BET) and zeta potential. The resultant MNA-7 h milling time displayed a crystalline structure with irregular shapes of 11.23 nm, specific surface area of 5.98 m^2^g^−1^, saturation magnetization, *Ms* of 8.35 emug^−1^, and isoelectric point charge at pH 5.4. The optimum adsorption capacity, *q*_*e*_ of 4.42 mg.g^−1^ for the removal of Cu^2+^ ions was attained at 120 min of contact time. The experimental data were best fitted to the Temkin isotherm model. A comparison between experimental kinetic studies and the theoretical aspects showed that the pseudo-second-order matched the experimental trends with a correlation coefficient of (*R*^2^ > 0.99). Besides, regeneration efficiency of 70.87% was achieved after three cycles of reusability studies. The MNA offers a practical, efficient, low-cost approach to reutilize mill scale waste products and provide ultra-fast separation to remove Cu^2+^ from water.

## Introduction

Water is one of the most valuable natural resource-based in nature and plays a fundamental role in the existence of all living beings [1]. With the revolution of the worldwide economy and the speedy expansion of contemporary industrialization, the challenges of water pollution become more severe [2]. The noxiousness of the ecological environment through heavy metals is intensely growing all over the world due to the enormous number of discharges emanating from industrial and population routes. The non-biodegradable heavy metals are very poisonous and have a tendency to build up in human being organs and other dwelling organisms, undermine the water quality supply, trigger various difficulties on aquatic life and cause numerous illnesses and disorders [3–5]. In particular, the release of wastewaters comprising copper(II) ions (Cu^2+^) resulting from anthropogenic pathways into domestic waters has turned out to be problematic for public health and environment [6–8]. Though Cu^2+^ is an important trace element in the human bodies and various biological routes and metabolism of animals [9], enormous consumption at elevated concentrations could result to severe toxicological impacts, for example convulsions, cramps, vomiting, or at the same time death. For instance, contamination due to divalent Cu initiates dramatization, keratinization, prickling of hands and feet, and exhibits carcinogenic and mutagenic influences [10–12]. Besides, the superfluous consumption of copper will provoke oxidative trauma and acute neurodegenerative syndromes, including amyotrophic lateral sclerosis, Menkes disorder, Alzheimer's illness and Wilson disease [13, 14]. The pollution of water by Cu^2+^ is regarded as one of the most prevalent environmental nuisance since Cu^2+^ compounds are contained in glut of industrial activities [15]. Conversely, the deficiency of Cu^2+^ in animal nutrition may cause diarrhoea, anaemia, and nervous disorders [16]. The World Health Organization (WHO), stipulated that the tolerable level for Cu^2+^ in drinking water is at 2 mgL^−1^ [17, 18]. Also, the United States Environmental Protection Agency (USEPA) fixed the maximum acceptable concentration of copper in the water to be 1.3 mgL^−1^ [19]. Thus, the level of Cu^2+^ in daily diet, particularly in drinking water and wastewater, should be monitored and lowered to a smallest amount prior to release into the surroundings [20].

In an attempt to curb these hazardous effects on the environment, numerous effluent remediation methods have been utilized such as precipitation [21], ion exchange [22], chemical precipitation, co-precipitation [23], membrane processes [24, 25, 82], coagulation [26], and adsorption [3, 27–30] to remove Cu^2+^ from wastewater. Amidst these approaches, adsorption is strongly desirable owing to its extremely convenience, cost-effective, ease of operation, flexibility, simple design procedure, simplicity, superior removal efficacy, wider practicality and recyclability [10, 31–33]. Hence, the central focus has been shifted to exploiting new adsorbents comprising diverse functional groups which could accelerates Cu^2+^ removal.

Several adsorbents can be applied to remove heavy metals from water via adsorption, including activated carbon [34], agricultural biomass [35], metal oxides [36], silica nano-materials [37, 38], clay minerals [31, 39, 40], others [41]. Yet, more applications of these sorbents are restricted due to their complex condition or modification of specific equipment and low adsorption. Furthermore, conventional adsorbents show weak recovery of the target metal ions from large bulks of solution owing to diffusion inadequacies, minimal binding capacity, and the insufficiency of active surface sites [42]. Therefore, the need to explore low-cost novel nano-sorbent with higher adsorption efficacy, substantial adsorptive surface area, minimal diffusion resistance, superior adsorption capacity and rapid separation for enormous volumes of solution is indispensable.

Recently, various new absorbents have been utilized, including nano-materials, mesoporous materials, carbon nanotubes (CNT), ion-coated materials, and magnetic nanoparticles [11, 43–45]. Among these, nano-based materials have received great attention due to their biological, physical and chemical characteristics, consequence of the large surface-to-volume ratio, higher absorption of metal ions with a superior adsorption capacity [46–48] The magnetic nano-sorbent has strong tendency to adsorb contaminants from gaseous or aqueous liquid waste, and the application of these magnetic sorbents to unravel many environmental contamination setbacks has gained increasing attention in recent years [11]. The magnetite adsorbent is a promising low-cost precursor with several unique properties such as their high surface area, superparamagnetic, high anisotropy, high coercivity, highly active low Curie temperature, ease of separation, high magnetic susceptibility, excellent recycling and reuse capabilities, magnetic attraction properties, among others [49, 50, 83]. Besides, the NPs are considered to be efficient for sorption of some metal ions, anions, ligands, cations and dyes, and their application is therefore attractive in an innovative field of adsorption, recovery or elimination of some ions [51–53].

In the light of this, it can be inferred that the studies using magnetite nano-adsorbent from a locally obtainable industrial milled chips using high-energy ball milling technique to remove Cu^2+^ from water are rather limited and it has been scarcely applied in spite of its impressive potential. In this regard, this study focuses on the synthesis of novel magnetite nano-adsorbent (MNA) from mill scale waste using high-energy ball milling method and its application as an economical removal of heavy metals copper (II) ions in water, from model of an aqueous solutions. Furthermore, surface kinetic study of the MNA recycled from mill scale waste used as metal adsorbent in wastewater treatment was also investigated. The kinetic model equation of MNA reaction was carefully developed and studied.

## Materials and Methods

### Materials and Chemicals

In this work, the raw mill scale waste chips were supply from steel factory located at Terengganu, Malaysia. Deionized water (DI) used in the batch experiment was obtained from the refinement system Milli-Q water. The copper nitrate (Cu(NO_3_)_2_) was procured from Aldrich (Chemical Industry Stock Co., Ltd., China) for the preparation of copper standard solution (1000 ppm). A pH 5-SS (spear pH Tester) was employed in the experiment for pH measurement and stirring. The UV–Vis spectrophotometer (HACH DR4000U) was utilized to analyse the copper concentration at 600 nm wavelength. The initial copper concentration was measured using the standard procedure (APHA, 2005) [54], and the remaining bulk of the sample was preserved in a chiller at 4 ℃.

### Synthesis of Magnetite Nano-Adsorbent (MNA)

**Fig. 1:**
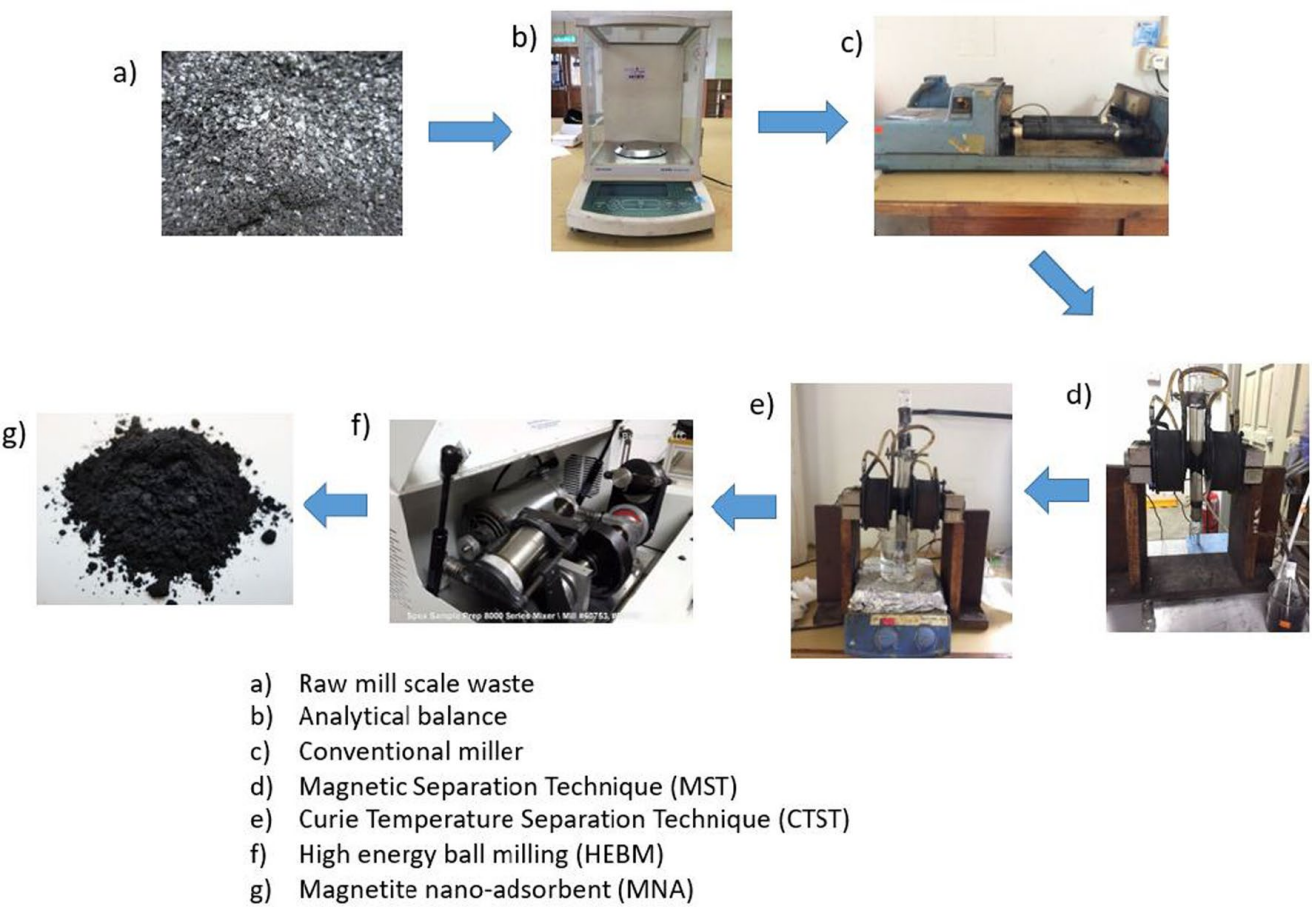
Synthesis procedure of magnetite nano-adsorbent using HEBM method

The raw mill scale waste chips contain magnetic particles and impurities (non-magnetic particles). The impurities were expunged to prevent sample contamination. Figure 1 describes the method used for the synthesis of magnetite nano-adsorbent (MNA) from milled chips. Firstly, the milled chips were extensively washed using DI water and dried at 104 ℃ for 24 h and then crushed into micron-size using a conventional milling machine. This procedure was steadily undertaken for 48 h, and the resultant micro-sized magnetite (Fe_3_O_4_) was subsequently cleansed with magnetic separation technique (MST). The MST stimulate the separation of non-magnetic and magnetic particles. Afterward, the cleaned micron magnetite was oven-dried for 48 h at 104 ℃ and later conveyed into an airtight container. Also, the strong magnetic particles were separated from the weak ones by a Curie temperature separation technique (CTST), as indicated in Fig. 1. This method is according to the procedure adopted by [55–57]. The separated robust magnetic particles were subsequently air-dried for 24 h and then subjected to a high-energy ball milling (HEBM) for three different milling times of 3, 5 and 7 h to attain a nano-sized magnetite [58].

### Characterization of Prepared Nano-Magnetite Adsorbent (MNA)

The structural morphology of the synthesized adsorbent was analysed with TEM/EDS using Hitachi Co., Japan Model No. S3400N. The FTIR provide information on functional groups existing on the synthesized adsorbent. The Bruker-Tensor 27 IR appliance with standard KBr-pellet method in the spectral range 400–4000 cm^−1^ with 2 cm^−1^ resolution identified FTIR spectra of the nano-magnetite adsorbent. The X-ray diffraction (XRD) technique was applied to analyse the crystal structure and phase of the synthesized MNA using the X-ray diffraction (XRD) Philips Expert Diffractometer with Cu Kα radiation (λ = 0.154 nm) obtained in the 2θ range of 20 to 80° with a scan step size of 2θ = 0.033 with 5 s per step as the counting. The observed XRD spectrum was compared with the standard ICSD database. The structural and morphological compositions of the MNA sample were obtained using a field emission scanning electron micrograph (FESEM), JEM JEOL 2100, USA, with high-resolution transmission electron microscope (HRTEM). The Brunauer–Emmett–Teller (BET): Micromeritics II PLUS, USA, was conducted to identify the specific surface area of the MNA by nitrogen adsorption–desorption using NOVA2020e automatic surface area and porosity analyser. Before the analysis, the MNA was degassed at 100 °C. The zeta potential measurements were conducted using a zeta sizer (Malvern ZS, UK). The zeta sizer provided a titration of several pH values. The investigation on the magnetic properties of the MNA powdered sample was done using a vibrating sample magnetometer (VSM) Model: LAKESHORE 7404, with an applied external field of 0–13 kOe (kG).

### Adsorption Studies

The sorption tests were performed using batch system. A stock solution of copper nitrate (Cu(NO_3_)_2_) (50 mgL^−1^) was prepared and diluted to give the suitable concentrations. The pH was adjusted using 0.1 molL^−1^ HCl or 0.1 molL^−1^ NaOH. The initial and final concentrations of Cu^2+^ were determined using an ultraviolet–visible spectrophotometer (UV–Vis) (Model: HACH DR4000U) at λ = 600 nm, based on prepared calibration curve. All sorption tests were performed using 250 mL flasks, to which an appropriate quantity of adsorbent and 100 mL of the ion solution were added. The kinetic studies were performed using 1 mg/L initial concentration of Cu (II) ion with 0.5 g of Fe_3_O_4_. The temperature and pH were kept constant at 25 °C and pH 7, respectively. The initial concentrations of Cu (II) ions were 10, 20, 30, 40, 50 mg/L in 200 mL. The pH was varied at 2, 4 6, 8, 10 and 12 by adding HCl and NaOH. Preliminary studies demonstrate that the adsorption process reached an equilibrium condition in 180 min. To study the dosage that influenced the adsorption capacities, different MNA dosage, ranging between 10 and 50 mg, was used. The percentage removal (%*RE*) and adsorption capacity (*q*_e_) of Cu^2+^ ions were determined using Eqs. 1 and 2, respectively [59, 60].

1 $$\% RE = \frac{{C_{o} - C_{e} }}{{C_{o} }} \times 100$$

2 $$q_{e} = \frac{V}{m}\left( {C_{o} - C_{e} } \right)$$

where *C*_*o*_ and *C*_*e*_, represented the initial and final concentrations (mgL^−1^) of the solution, respectively. *V* is the volume of the solution in litres, and *m* is the mass of the adsorbent in grams (g). *q*_*e*_ (mg/g) is the amount of adsorbate per unit mass of adsorbent at time *t.*

### Kinetic Study

The experiments were conducted using Jar Tester with 200 mL copper solution at a constant temperature of 28 ℃ and at pH 5.4. The samples were taken at a different time interval at 0, 10, 20, 30, 40 and 50 min and analysed using UV–Vis. The kinetic models were studied using Lagergren pseudo-first-order and pseudo-second-order. Lagergren’s pseudo-first-order is depicted in Eq. (3):

3 $$q_{t} = q_{e} \left( {1 - e^{{ - k_{1} t}} } \right)$$

where *q*_e_ (mg/g) and *q*_t_ (mg/g) are the amounts of adsorbed adsorbate at equilibrium and at time *t*, respectively, and *k*_1_ (min^−1^) is the rate constant of pseudo-first-order adsorption. For rate of adsorption in second-order mechanism, the pseudo-second-order kinetic rate equation can be expressed as Eq. (4):

4 $$q_{t} = \frac{t}{{\frac{1}{{k_{2} q_{e}^{2} }} + \frac{t}{{q_{t} }}}}$$

where *k*_*2*_ (gmg^−1^.min^−1^) is the equilibrium rate constant of pseudo-second-order adsorption.

### Regeneration Study

The MNA was re-claimed by solvent desorption approach when the active pore sites attained equilibrium. The MNA was separated by an external magnet from aqueous solution, and subsequently dipping into HCL solution and mixing for 180 min at 26 ℃. The resultant MNA was then rinsed with distilled water to achieve neutral pH and then maintained for 1 h at 60 ℃. The regenerated MNA was then re-used in tandem with previous studies [54, 55]. The re-usability efficiency (RE%) was calculated using Eq. 5:

5 $$RE = \frac{{q_{reg} }}{{q_{ori} }} \times 100\%$$

where *q*_reg_ and *q*_ori_ are their respective adsorption capacities per unit mass of the regenerated and original adsorbent.

### Statistical Analysis

The experimental data were subjected to a fully randomized design, and the data obtained were analysed using one-way analysis of variance (ANOVA) by a general linear model (GLM) procedure in SAS software 9.4 Version (SAS Institute Inc., Cary, NC, USA). Duncan multiple range test was used to separate means at *p* < 0.05 significance level.

## Results and Discussion

### Structural and Phase Analysis

**Fig. 2:**
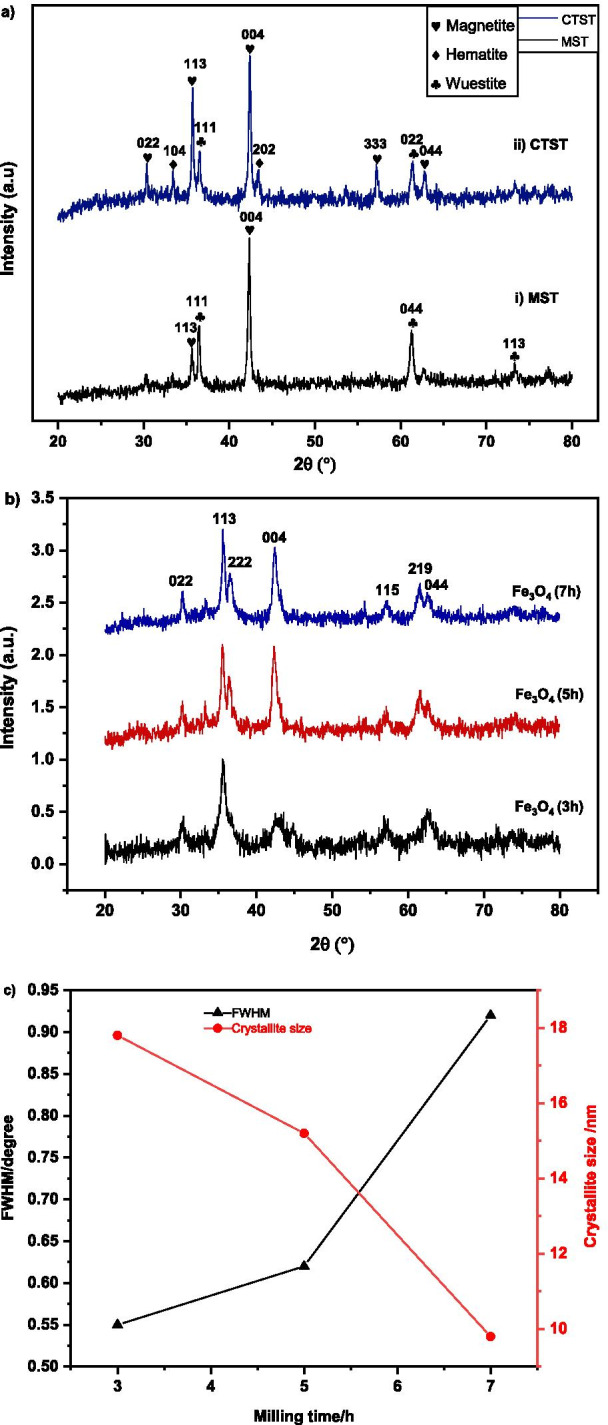
**a** X-ray diffraction spectra of mill scale after undergoing MST and CTST, **b** indexed spectra of magnetite at different milling times of 3, 5, and 7 h, **c** The full width at half maximum (FWHM) and crystallite size of MNA with milling time

The results from XRD examination of waste mill scales after MST and CTST are shown in Fig. 2a. After MST process (Fig. 2ai), the XRD confirmed the presence of wuestite (FeO) and magnetite (Fe_3_O_4_). The Bragg diffraction of wuestite was observed at 2θ of 36.33° (111), 61.40° (044), 73.25° (113) as matched to ICSD: 98–001-2335. The magnetite phase was observed at 2θ of 35.61° (113) and 43.28° (004), agreed to reference Fe_3_O_4_ ICSD file 98–010-9826.

For XRD spectrum after CTST process (Fig. 2aii), it was observed the presence of magnetite, hematite and wuestite phase. The Bragg diffraction angle (2θ) of all the peaks was essential identify and agreed to the reference ICSD file 98–010-9826 for magnetite with the peaks are 30.23° (022), 35.61° (113), 43.28° (004), 57.24°(333), 62.86° (044). The hematite peaks also observed at 33.44° (104), 48.48° (202) as matched to ICSD 98–004-6407); and wuestite diffraction angles of 36.33° (111) and 61.27° (022) as matched to ICSD: 98–001-2335. The result was agreed and similar as reported in the previous literature [84].

Figure 2b illustrates the XRD diffraction patterns of mill scales powder after high-energy ball milling process, at different time intervals of 3, 5, and 7 h. The diffraction spectra of the synthesized sample revealed the presence of magnetite (Fe_3_O_4_) phase with nano-sized in diameter for all milling times. The diffraction angle at 2θ of 30.24°, 36.66°, 36.57°, 42.45°, 57.26°, 61.54° and 62.76° can be indexed to (022), (113), (222), (004), (224), (115) and (044) confirming the signature peaks of a cubic unit cell Fe_3_O_4_, respectively. The XRD spectra were matched to the reference ICSD 98–01-11,241 of magnetite with a space group of *Fd*-3* m* and lattice parameter (*a* = *b* = *c*) of 8.3440 Å. The nano-magnetite adsorbent exhibits high purity with increase in the milling time as demonstrated in Fig. 2b. High energy generated by the colliding steel ball in the vials is responsible for breaking the oxygen bond and reduces the hematite (Fe_2_O_3_) to the magnetite (Fe_3_O_4_) phase. As milling time increases, it was observed the formation of nanocrystalline magnetite was determined by the broadening of XRD peaks. As increase milling time, the XRD peak broadening was observed to be increased, indicating the decreases of the particles size. The XRD peak intensities were also observed to decrease, with increasing the milling time. The pattern is indicating the decrease in the particle size of the samples [61]. As the particle size decreases, the strains induced during the milling process resulted in a decrease in the peak intensity and broadening of the diffraction peak. The average crystallite size *D* of samples was calculated using the Debye–Scherrer formula as in Eq. (6) [62].

6 $$D = \frac{0.9\lambda }{{\beta \cos \theta }}$$

where *D* is the average crystallite size, *λ* is the wavelength of X-ray (0.1541 nm), *β* is the full width at half maximum (FWHM), and *θ* is the diffraction angle. The XRD spectra were automatically analysed using X’pert Highscore Plus software. The relation of FWHM and crystallite sizes are shown in Fig. 2c. The analysis shows that the change in the microscopic MNA powder with the increase in the milling time from 3, 5 and 7 h with the variation change in the FWHM and the average crystallite size of MNA as shown in Fig. 2c. The variation trend of FWHM revealed that with the increase in the milling time from 3, 5 and 7 h, the FWHM shows an increase trend. With the increase in milling time from 3, 5 and 7 h, the average crystallite size decreased from a minimum value of 17.8 nm, 15.2 nm and 9.8 nm, respectively.

### Morphological and Microstructural Composition

**Fig. 3:**
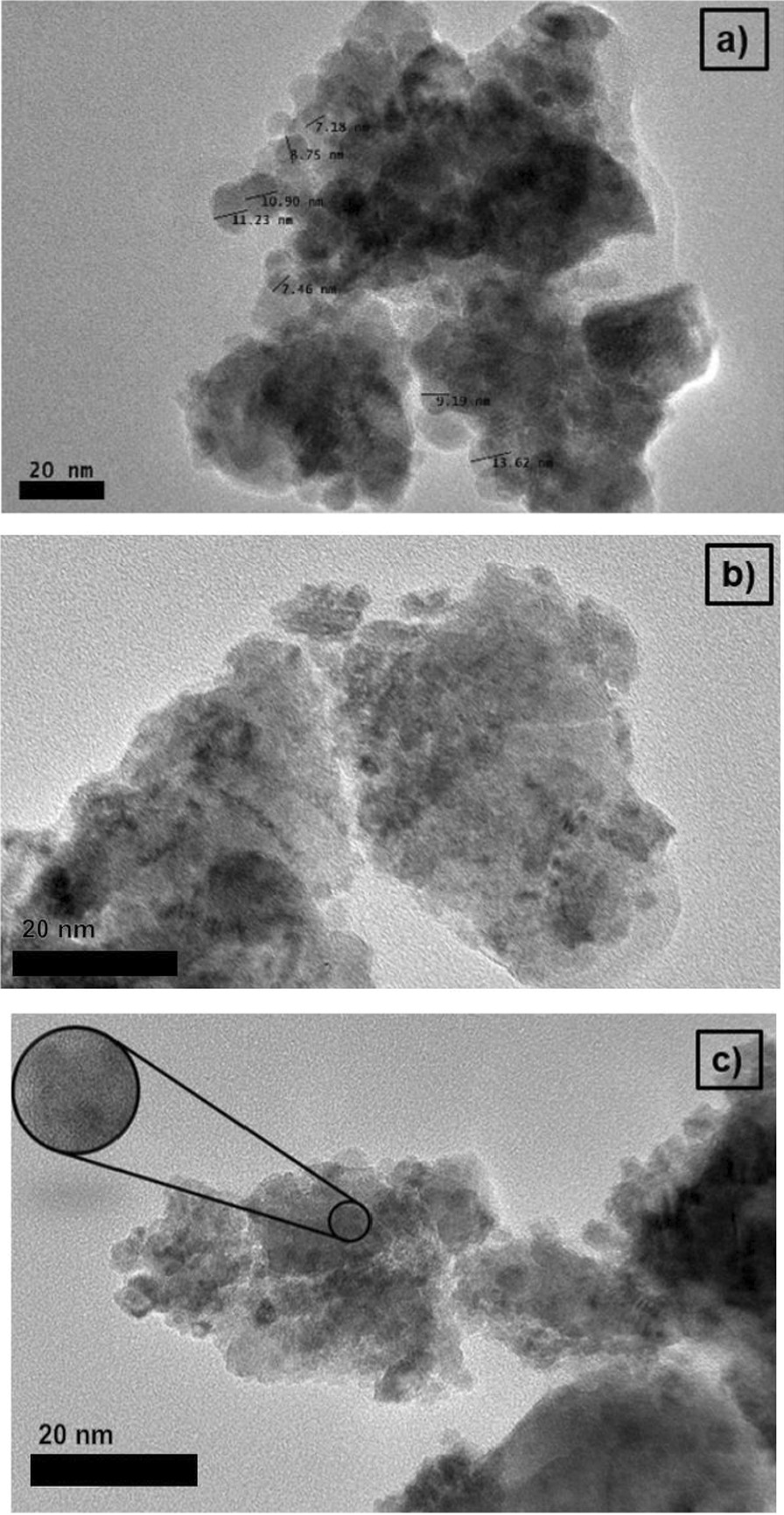
HRTEM images with a 20-nm scale bar of MNA at **a** 3 h **b** 5 h and **c** 7 h milling time

The HRTEM micrographs of the MNA milled at different milling time of 3, 5 and 7 h are presented in Fig. 3. The micrographs show that the MNA particles exhibit an irregular shape during the three milling periods as shown in Fig. 3. Also, an average MNA particle size of 5.53 nm was noticed at 3 h milling time as compared with the 14.45 nm (5 h) and 19.16 nm (7 h). This implies that smaller MNA particle size could be achieved at shorter milling time. The average particle size for 3, 5 and 7 h was obtained in the range of 10 to 22 nm. As the milling time increases, the micro-strain in the sample also increases [63]. Hence, prolonged milling hours will produce more strain to the samples. The rise in lattice strain with milling time was due to a strong distortion effect caused by atom dislocation and diffusion in the lattice introduced during the milling process. However, agglomeration effects were observed in the MNA sample as shown in Fig. 3, due to the magnetic attraction behaviour of the magnetite powder.

### Magnetic Properties Analysis

**Fig. 4:**
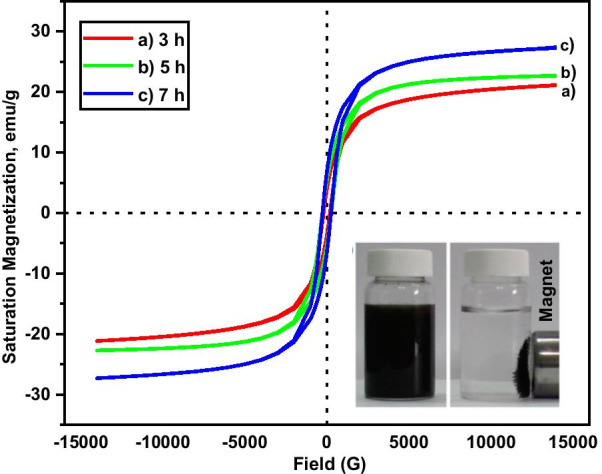
*M-H* hysteresis graph of samples at different milling times **a** 3 h, **b** 5 h and **c** 7 h

**Fig. 5:**
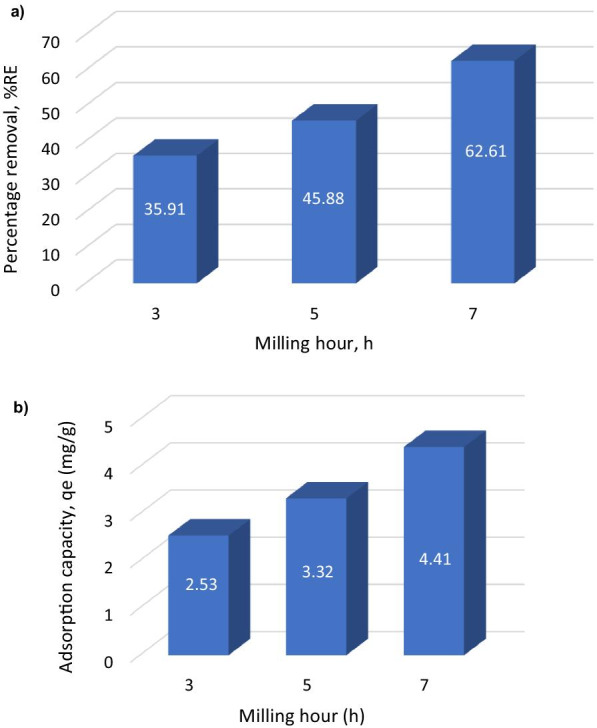
**a** The bar chart of percentage removal; **b** metal uptake/ adsorption capacity of MNP onto Cu^2+^ at different milling hours

The magnetic properties of the samples were investigated using a VSM at room temperature experiment. The response of magnetization (*M*) with the applied external magnetic field (*H*) of the samples is shown in Fig. 4. The saturation magnetization (*M*_s_), remanence (*M*_r_) and the coercivity (*H*_c_) of the samples are summarized in Table 1. The coercivity value of the MNA is in the range of 200–270 G, the remanence between 1.5 and 6.6 emu/g, and the saturation magnetization values between 21 and 27 emu/g. Due to the diameter of the particle size which is less than 20 nm, the samples have a superparamagnetic property. From the magnetization parameters (Table 1), this indicates that the samples consist of a mixture of the superparamagnetic and ferromagnetic compounds that contribute to the increase in adsorption capacity. The MNA-7 h sample shows the highest magnetic parameters (Fig. 4) that contributed to the highest adsorption capacity from Cu adsorption study (Fig. 5).

**Table 1:** The remanence (*M*_r_), saturation magnetization (*M*_s_), coercivity (*H*_c_) and TEM particle size of MNA nano-absorbent

Sample (h)	Remanence, *M*_*r*_(emu/g)	Saturation magnetization, *M*_*s*_(emu/g)	Coercivity, *Hc* (G)	Approximation particle size by HRTEM (nm)
3	1.53	21.12	199.99	16.54
5	5.44	22.69	270.24	15.98
7	6.61	27.34	262.18	11.23

### Effect of Adsorption Parameters

Further analysis on the batch adsorption study of MNA of 3, 5 and 7 h has been investigated. Figure 5 shows the absorption study was carried out for the MNA at various milling time of 3, 5 and 7 h. The graph shows the highest adsorption capacity (metal up-taken) (*q*_e_) and highest percentage removal (%*RE*). The MNA-7 h milling hours shows the highest adsorption capacity and highest percentage of removal from aqueous solution. Therefore, MNA-7 h was chosen as the MNA nano-absorbent for further batch absorption analysis on several parameters of contact time, initial concentration, absorbent dosage, the surface area, pH and temperatures.

### Surface Area Analysis

**Fig. 6:**
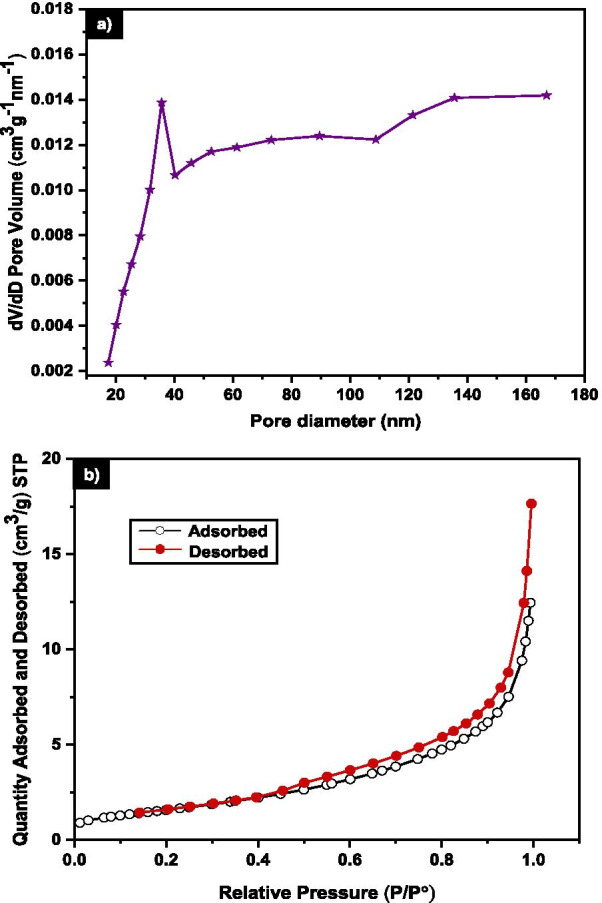
**a** Distribution of pore diameter of 7 h MNA **b** Nitrogen adsorption–desorption isotherm

The nitrogen adsorption using BET was utilized to assess the surface area and pores characteristics of MNA for 7 h milling. Figure 6a shows the BET result for MNA-7 h with average pore volume of 0.011 cm^3^g^−1^ and specific surface area of 5.98 m^2^g^−1^. The N_2_ adsorption–desorption curve of MNA-7 h lies in the Type III hysteresis curve (Fig. 6b) as agreed with previously reported by Sing et. al. (1985) [65]. The BET results describe the adsorbent–adsorbate interactions of that adsorbed molecules are clustered around on the surface of the MNA [66]. Hence, the adsorption in type III reveals the gas molecules were physically adsorbed onto MNA [67].

### EDS Analysis

**Fig. 7:**
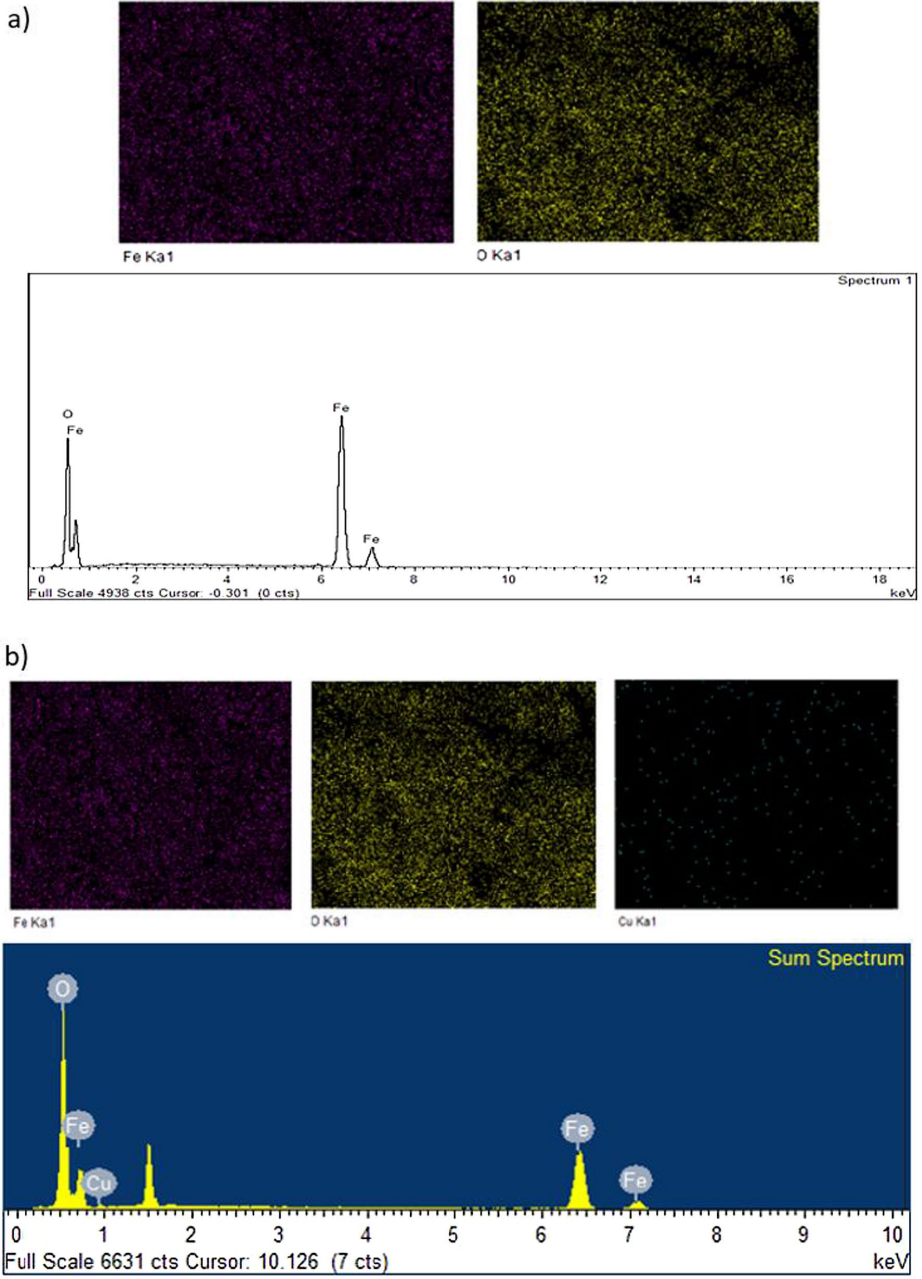
EDS spectrum analysis (**a**) before the adsorption and (**b**) after adsorption of Cu (II) ions

The elemental constituents of MNA-7 h are illustrated in Fig. 7. The FESEM and EDS analyses revealed the presence of element Fe and O with percentage of 78.25% and 21.75%, respectively, for MNA-7 h before adsorption process (Fig. 7a). Figure 7b depicts the EDS spectra that the presence of element Fe, Cu and O after adsorption process. The existence of copper on the spectra shows the adsorption of Cu^2+^ by MNA. This trend also is in agreement with the spectra displayed in the studies reported by Lingamdinne et al. (2016) [64] as the iron oxide nanoparticles was utilized for the adsorptive removal of heavy metals.

### FTIR Analysis

**Fig. 8:**
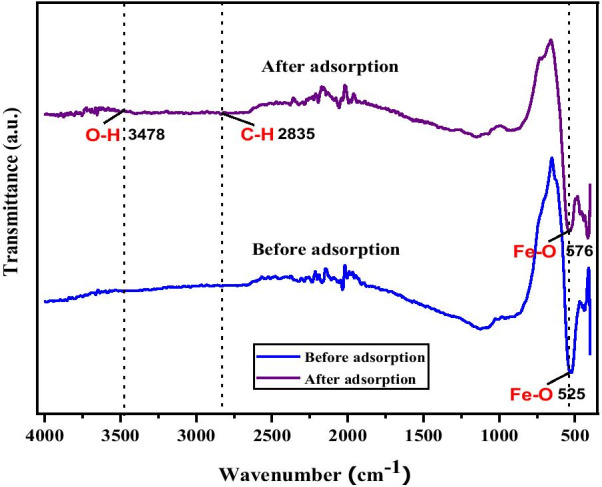
The Fourier transform infrared spectra of MNA-7 h before and after adsorption of Cu (II) ion

Further confirmation of Cu adsorption onto MNA-7 h was also determined via a FTIR analysis. The FTIR was investigated to identify the functional group and attachment of Cu^2+^ onto MNA. Figure 8 shows the FTIR spectra of the MNA-7 h before and after adsorption of Cu (II) ions. The FTIR spectra reveals a strong characteristics peak band of the Fe_3_O_4_ nanoparticles. After the adsorption, the FTIR spectrum reveals the changes in bands intensity in the range of 500–600 cm^−1^ and 2800–3600 cm^−1^ that leads to Cu^2+^ sorption. Besides, the adsorption bands at 525 cm^−1^ and 576 cm^−1^ represent the tetrahedral and octahedral sites of the Fe–O band magnetite nanoparticles [68]. The strong and broad adsorption spectrum at 3478 cm^−1^ corresponds to the hydroxyl group (− OH) and traces of water molecule on the surface of MNA [69]. The FTIR spectroscopy reveals that the MNA has a crystalline structure due to the presence of a few chemical substances adsorbed on the surface of the MNA.

### Zeta Potential Analysis

**Fig. 9:**
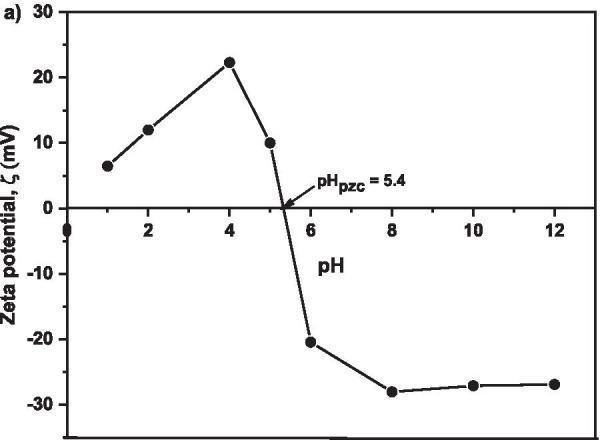
Zeta potential for pH values of 1, 2, 4, 6, 8, 10, and 12 for MNA-7 h

Figure 9 shows the surface zeta potential of MNA-7 h. Zeta potential was investigated to get an accurate and precise determination of the neutralization charge occurred from the adsorption process. Zeta potential data provide a measurable value to monitor the optimal adsorbent dosage during Cu adsorption process. The results shows that the isoelectric point (pH_pzc_) occurred at pH 5.4, the point at which Cu^2+^ adsorption onto MNA occurred is at optimum. In an aqueous solution, the surface of iron oxides is covered with OH^−^ group, such that FeOH on the surface could change to other Fe functional groups such as FeO or FeOH_2_, due to the protonation or deprotonation process [70, 71]. The balance of protonation and deprotonation depend on the pH of the solution, and the pH_pzc_ of the absorbent. The zeta potential results suggest that the adsorption was efficient at pH of 5.4.

### Batch Adsorption Analyses

#### Effect of Contact Time

**Fig. 10:**
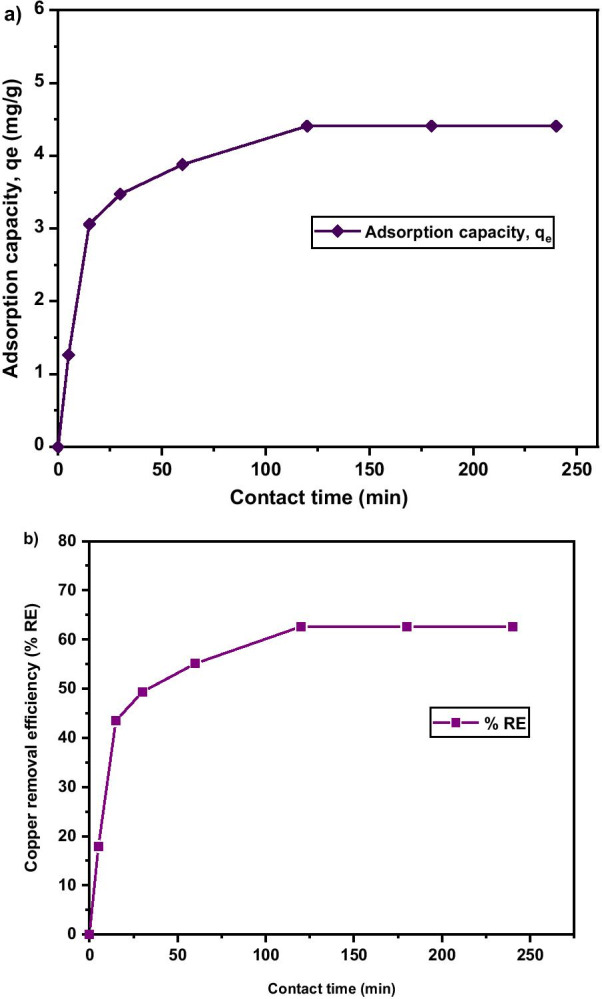
**a** Adsorption capacity; **b** Copper removal efficiency under various contact times of MNA-7 h (metal solution: 200 mL; temperature: 25 °C; initial pH: 5.4; initial concentration: 50 mg/L; adsorbent dosage: 0.05 g)

Figure 10a shows the effect of contact time on adsorption capacity and rate of Cu^2+^ uptake onto MNA after 250 min. It is evident that at longer contact time, the adsorption capacity reach equilibrium as the pH was kept at 5.4 and adsorbent dosage at 0.05 g. The maximum removal efficiency attained was 62.61% as shown in Fig. 10b. The Cu^2+^ removal efficiency surges rapidly from the early first 5 min, and later slower and stable throughout the adsorption process. This is attributed to the fact that the rate of the adsorption capacity was high due to the abundant free binding and active sites of the Cu^2+^. Based on Fig. 10, it was noticed that the percentage removal and adsorption capacity increased rapidly with the increase in contact time at the initial stage. The contact time has a substantial influence on the efficacy of Cu^2+^ removal and adsorption capacity. Increase in contact time from 0 to 240 min led to an increase in the removal efficiency of Cu^2+^ from 0.81% to 62.61%. For contact time greater than 120 min, the removal efficiency of Cu^2+^ remains steady, as the active sites has been saturated on the surface of the adsorbent. Similarly, the highest adsorption capacity of 4.41 mg/g was attained at 120 min of contact time. Thus, the equilibrium time was attained at 120 min.

The results of copper removal efficiency follow a definite trend (Table 2). It shows that the higher the time, the more the removal efficiency. There were significant differences (*p* < 0.05) among the removal efficiencies under varying contact times. Generally, as contact time progresses, the removal efficacy also improves. Table 3 shows the percentage of the copper removal at different adsorbent dosages. The result shows that as the time progresses, the removal percentage increases. 0.05 g adsorbent dosage recorded the highest copper removal efficiency (62.58 g) after 120 min contact time.

**Table 2:** Copper removal efficiency at varied contact time

Time	Removal efficiency
0	0.00 ± 0.00^f^
5	17.50 ± 0.21^e^
15	43.04 ± 0.32^d^
30	48.99 ± 0.23^c^
60	55.03 ± 0.07^b^
120	62.31 ± 0.18^a^
180	62.62 ± 0.01^a^
240	62.45 ± 0.08^a^
*p *value	< .0001

The letters indicate the level of significant and differences in the removal efficiencies of Cu2^+^

**Table 3:** Copper removal efficiency under different dosages (0.05–0.08 g) The letters difference a-g indicate the level of significant differences in copper removal efficiencies at varied dosage (p<0.0001).

Time	Dosage			
	0.05 g	0.2 g	0.5 g	0.8 g
0	0.00^f^	0.00^f^	0.00^ g^	0.00^ g^
5	17.67^e^	30.56^d^	7.15^f^	7.03^f^
15	43.18^d^	31.27^c^	11.34^e^	8.46^e^
30	49.30^c^	36.52^b^	14.31^d^	10.07^d^
60	55.10^b^	37.27^a^	21.82^c^	11.56^c^
120	62.58^a^	37.44^a^	38.73^b^	13.26^b^
180	62.61^a^	37.43^a^	40.43^a^	24.31^a^
240	62.62^a^	37.23^a^	40.29^a^	24.30^a^
*p *value	< .0001	< .0001	< .0001	< .0001

The letters indicate the level of significant and differences in the removal efficiencies of Cu2^+^

#### Effect of Initial Concentration

**Fig. 11:**
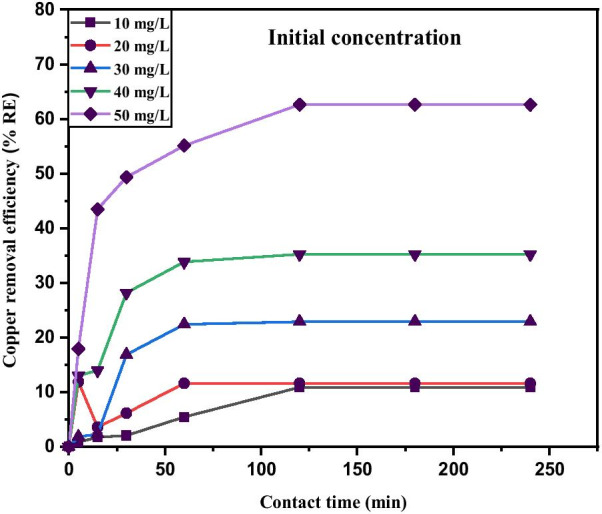
Copper removal efficiency under various initial concentration of MNA-7 h (metal solution: 200 mL; temperature: 25 °C; initial pH: 5.4; adsorbent dosage: 0.05 g)

Figure 11 shows as the initial concentration increases, the equilibrium adsorption capacity also increases. Thus, the higher initial concentration due to 0.05 g adsorbent in the Cu^2+^ solution to fill the active sites on the adsorbent and the quantity of copper adsorbed increases with the increase in Cu^2+^ concentration [83]. The initial concentration of Cu^2+^ increased from 10 mg/L to 50 mg/L with corresponding increase in adsorption capacity from 0.04 mg/g to 4.41 mg/g, which in turn provide a higher driving force for the ions from the solution to the adsorbents, resulting in more collisions between Cu^2+^ and active sites on the MNA-7 h. Since nearly all the adsorption sites of MNA-7 h existed on their exterior, it is easy for the adsorbate to access these active sites, thereby facilitating a rapid attainment of equilibrium condition.

#### Effect of MNA Dosage

**Fig. 12:**
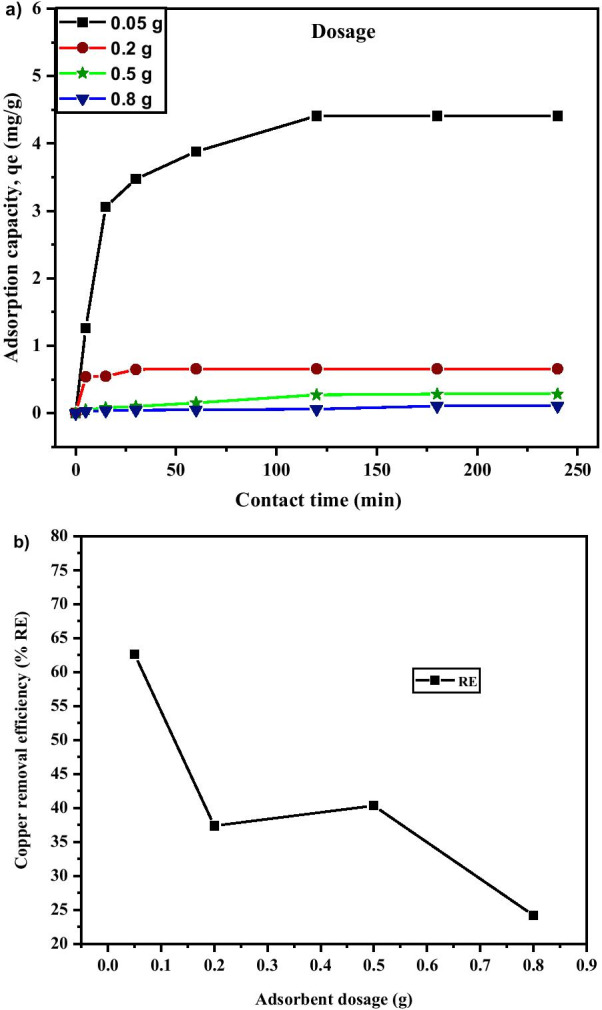
**a** Adsorption capacity; **b** Copper removal efficiency under various adsorbent dosage of MNA-7 h (metal solution: 200 mL; initial concentration: 50 mg/L; temperature: 25 °C; initial pH: 5.4)

Adsorbent dosage plays an important role during the adsorption process, as it controls the ability of the adsorbent for a given solution. The more the dosage, the more obtainable site for sorption to occur [67]. Figure 12a shows the adsorption capacity, *q*_*e*_ of Cu^2+^ with respect to different dosages of MNA-7 h at 0.05 g, 0.2 g, 0.5 g, and 0.8 g, respectively. The adsorption capacity was observed to be dependent on adsorbent dosage, which determines the availability of the active sites and the amount of the surface area for adsorption. This is due to the increase in surface area and the probability of collision and interaction between the particles of nano-adsorbent and Cu^2+^ [72]. As shown in Fig. 12b, at 0.05 g dosage, 62.61% copper removal efficiency and 4.41 mg/g of adsorption capacity were recorded. The Cu^2+^ removal increases sharply and becomes stable as the adsorbent dosage increases. As the adsorbent dosage increases, the larger surface interaction and the agglomeration effects develop. Thus, it causes a decrease in free specific area per unit mass of MNA surface, causing a reduction in contact surface with the adsorbate surface. This will lead to the decrease in *q*_*e*_and %*RE.* Besides, the decrease in *q*_*e*_and %*RE*, perhaps was due to the saturation of Cu^2+^ in solution with respect to available adsorption binding sites [73]. Thus, a higher amount of adsorbent causes an aggregation which decreases the total surface area of the MNA, thereby leading to a decrease in adsorption capacity [74–76]. The aggregation could result to a decrease in total surface area of the adsorbent and an increase in diffusion path length [75].

#### Effect of pH

**Fig. 13:**
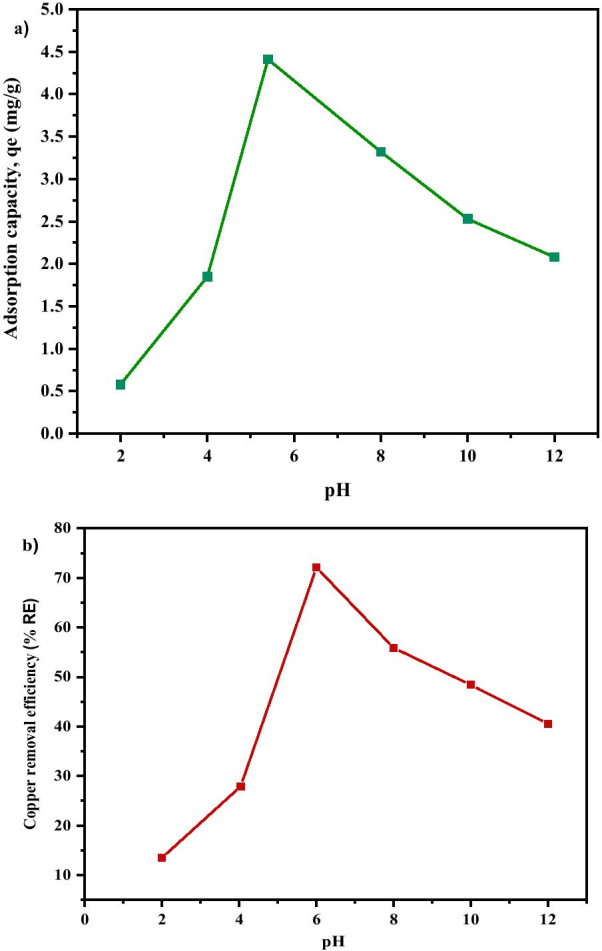
**a** Adsorption capacity; **b** copper removal efficiency under various pH of MNA-7 h (metal solution: 200 mL; initial concentration: 50 mg/L; temperature: 25 °C; adsorbent MNA dosage: 0.05 g)

The removal of Cu^2+^ from the aqueous solution through adsorption is highly dependent on the solution pH which determines the surface charge of the adsorbent and the adsorbate speciation [77]. Adsorption is regarded to be minimal at acidic state owing to higher concentration of H_3_O^+^ which competes with the positively charged ions for the actively binding site on the adsorbent surface, and this usually led to low contaminant removal [77, 78]. The influence of pH on the adsorption of Cu^2+^ on MNA was evaluated between the pH range of (2–12). Figure 13 shows the effect of pH on the adsorption capacity and removal efficiency of Cu^2+^. It was observed that increase in pH from 2 to 5.4, results in an increase in adsorption capacity from 0.58 mg/g to 4.408 mg/g and percentage removal of copper from 10.71% to 62.61%, respectively. However, the equilibrium adsorption capacity of Cu^2+^ is low at a strong acidic condition recording 0.58 mg/g at pH 2 due to the presence of a high percentage of H_3_O^+^ ion which competes with Cu^2+^ at the sorption sites of MNA. Besides, when pH is higher than 5.4, the adsorption capacity decreases from 49.32% to 44.69%. At a higher pH, higher concentration of OH^−^ causes a decrease in the adsorption rate. Figure 13a and b shows that pH has a significant impact on Cu^2+^ adsorption capacity and removal percentage (*%RE*) of Cu. The removal rate for Cu^2+^ increases with an increase in pH, from 10.71% to 28.04% and to 62.61% when pH is at 2, 4 and 5.4, respectively, before declining to 49.32%, 42.56% and 44.69 at pH 8, 10 and 12, respectively (Fig. 13a).

Similarly, the adsorption capacity also increases from 10.71% to 62.61% with the increase in pH from 2 to 5.4 and decreases until it reaches pH 12. At pH 2 and 4, the amount of protonation of the adsorbent surfaces results in a decrease in Cu^2+^ adsorption. The results also are in agreement with zeta potential graph as indicated in Fig. 9. Also, Fig. 9 describes the net charge of the MNA adsorbent surface at different pHs, with the point of zero charges (pH_pzc_). As the pH increases, the H^+^ ion is lower and causes the surface of the adsorbent to become negatively charged, with the increase in *%RE* of Cu^2+^, thereby increasing the electrostatic attraction force between the adsorbents in the solution [79]. Therefore, pH influences the surface zeta potential of MNA. The surface functionality of iron oxides varies depending on the nature of iron oxides and the pH value.

### Copper Adsorption Kinetics

**Fig. 14:**
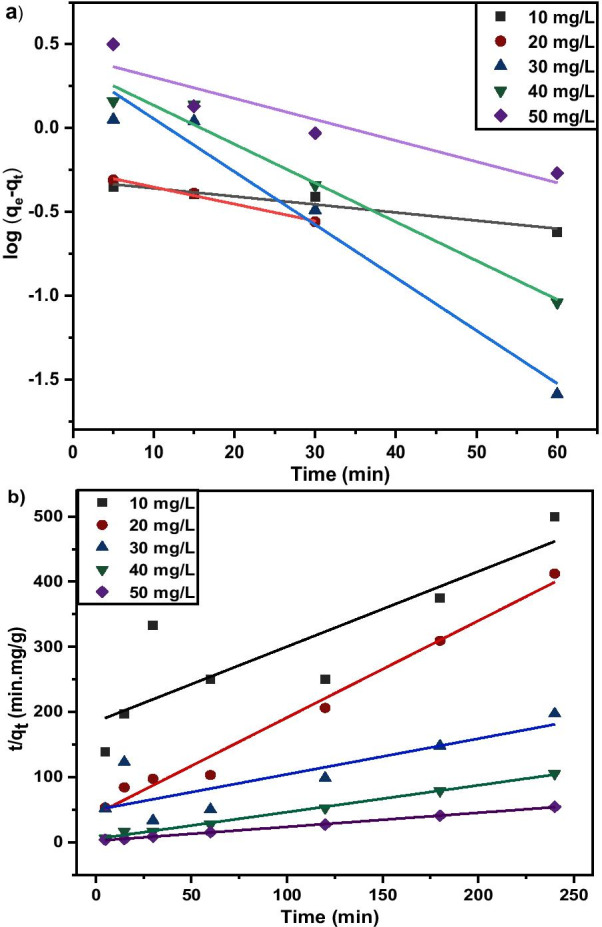
**a** Lagergren’s first-order kinetic model; **b** Pseudo-second-order kinetic model for the removal of Cu (II) ions at various initial concentrations from the aqueous solution using MNA at 0.05 g/L dosage of MNA-7 h

Kinetic studies are essential in the adsorption process to describe the uptake rate performance of MNA-7 h and influence the residual time for the entire adsorption process. The adsorption kinetics of Cu^2+^ on MNA-7 h was determined using similar procedures to those used in the batch adsorption studies [55]. The Lagergren’s first-order kinetic model and second-order kinetic model for the removal of Cu^2+^ at various initial concentrations from the aqueous solution using MNA at 0.05 g/L of the MNA dosage are shown in Fig. 14. The calculated *q*_e_ values are in agreement with the theoretical values, and the graph shows good linearity with *R*^2^ above 0.96. Therefore, the adsorption kinetics follows the pseudo-second-order model. The pseudo-second-order model represents the adsorption kinetics, involving donation or electron exchange between adsorbate and adsorbent. Table 4 shows the fitted parameter summary of Cu^2+^ kinetics at different initial Cu^2+^ concentrations (*q*_*e*_: mg/g, *k*_*1*_: min^−1^, *k*_*2*_: g/mg/min) of MNA-7 h. For the parameters of initial concentration, *C*_*i*_ (mg/L), adsorption capacity, *q*_*e*_ (mg.g^−1^), *k*_2_ is the rate constant of pseudo-first-order, *k*_2_ is rate constant of pseudo-second-order, and the *R*^2^ is the correlation coefficient. Two kinetic models: Lagergren’s first-order and pseudo-second-order order were applied to further study the rate of adsorption process for Cu^2+^. The kinetic parameters of pseudo-first-order and pseudo-second-order are presented in Table 4. According to Table 4, pseudo-second-order was best fitted for the adsorption of Cu^2+^. Pseudo-second-order revealed a higher correlation coefficient of *R*^2^ = 0.999, for Cu^2+^ removal.

**Table 4:** Adsorption rate constants and parameters of Cu (II) ion adsorption from the pseudo-first-order and pseudo-second-order kinetic models

Kinetic isotherm	Parameter	Initial concentration (mg/L)				
		10	20	30	40	50
Pseudo-first-order	*k*_*1*_(min^−1^)	0.1108	0.0233	0.0728	0.0534	0.0290
	*q*_*e, calc*_ (mg/g)	0.488	0.560	2.355	2.333	3.090
	*q*_*e, exp*_ (mg/g)	0.480	0.582	1.216	2.276	4.408
	*R*^*2*^	0.930	0.991	0.967	0.976	0.872
Pseudo-second-order	*k*_*2*_(min.mg/g)	0.0072	0.0514	0.0060	0.0327	0.0214
	*q*_*e, calc*_ (mg/g)	0.8667	0.6732	1.8268	2.4297	4.6350
	*q*_*e, exp*_ (mg/g)	0.480	0.582	1.216	2.276	4.408
	*R*^*2*^	0.740	0.984	0.680	0.995	0.999

### Copper Adsorption Isotherms

**Fig. 15:**
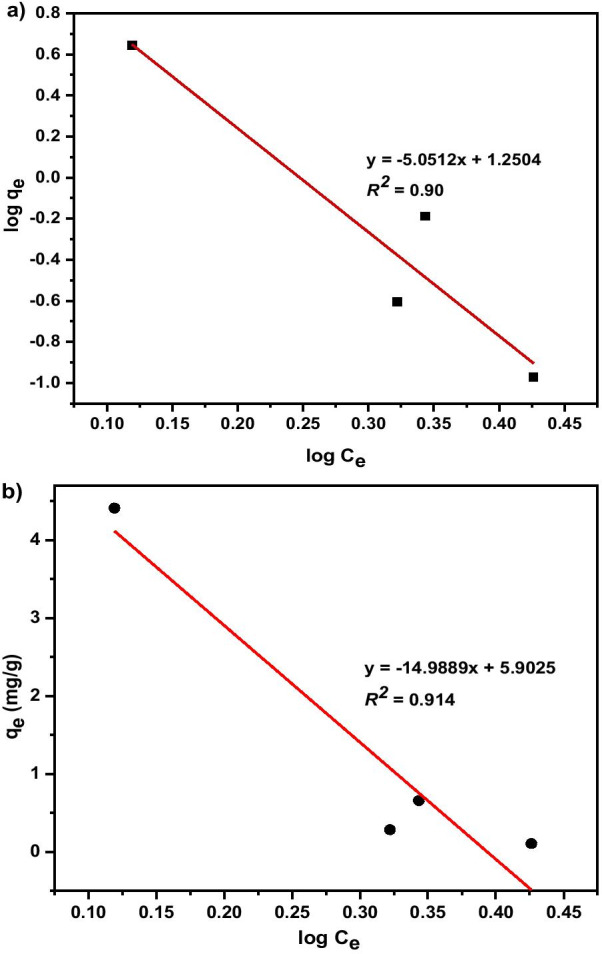
**a** Linearized Freundlich; **b** Linearized Temkin isotherm models for Cu^2+^ adsorption by MNA-7 h at various adsorbent dosages: contact time 240 min; initial pH 5.4; room temperature

The adsorption isotherms experimental data were investigated using Freundlich isotherm model (Eq. 7) and Temkin isotherm model (Eq. 8). The adsorption isotherm is illustrated in Fig. 15a and b. The Freundlich isotherm is expressed as:

7 $$q_{e = } K_{F} \times C_{e}^{1/n}$$

8 $$qe = B\log kt + B\log C_{e}$$

where *q*_*e*_ is adsorbent capacity at equilibrium (mg/g); *B*: (*RT*/*b*) is the Temkin constant related to heat of adsorption (J/mol) *R* is universal gas constant (8.314 J/mol K); *T* is absolute temperature (K); 1/*b* indicates the adsorption potential of the adsorbent; *kt* is equilibrium binding constant corresponding to the maximum binding energy (L/mg); and *Ce* is adsorbate concentration at equilibrium (mg/L). From the graph, the straight line emerged, and the values of *q*_*m*_ and *K*_*L*_ constants can be calculated using the slope and the intercept of the straight line. Freundlich and Temkin models was used to examine the relationship between the adsorbent and adsorbate. As shown in Fig. 15, the initial concentrate correlation coefficient with an *R*^*2*^ value of 0.914 and manifested Temkin adsorption isotherm to be more favourable for the removal of copper. The synopsis of the isotherm parameter of Freundlich, Temkin parameter with correlation coefficient, *R*^2^ for adsorption of Cu (II) on MNA-7 h at room temperature is depicted in Table 5. 1/*n* constant reciprocal implies natural sorption; therefore, the adsorption process is beneficial. The values of n, 1/*n*, *K*_F_, and *R*^2^ for the current work are also presented in Table 5. Temkin isotherm adsorption plot shows maximum Cu^2+^ removal by MNA-7 h at optimal conditions and reveals the feasibility of the process. The data prove that the Temkin model well fitted the experimental data than Freundlich based on the correlation coefficient, *R*^*2*^ (Table 5). This could be due to the fact that the Temkin isotherm model considers the effect of indirect adsorbate on the adsorption process and assumes that the heat of the adsorption of molecules decreases linearly in the adsorption layer [80].

**Table 5:** Freundlich, Temkin parameter with correlation coefficient, *R*^*2*^ for adsorption of Cu (II) on MNA-7 h at room temperature

Isotherm model	Parameter	Values
Freundlich isotherm model	*K*_*F*_	3.4917
	*1/n*	− 5.0512
	*R*^*2*^	0.90
Temkin isotherm model	*B*	− 14.9889
	*b*	151.5101
	k_t_	1.0940
	*R*^*2*^	0.91

### Regeneration and Desorption Study

**Fig. 16:**
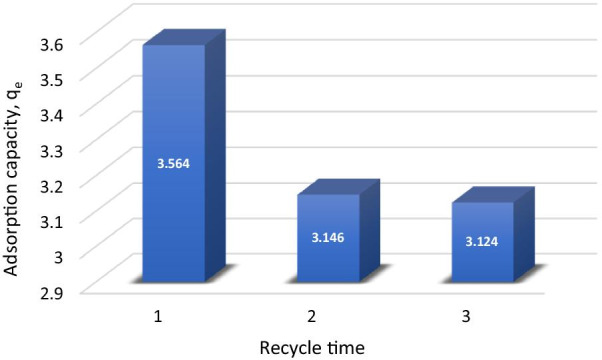
Desorption of copper onto MNA-7 h in three cycles

The reusability of adsorbent is a prime issue since periodically regenerating adsorbent is strongly desirable for industrial applications [81]. In the reusability tests, the adsorption and desorption cycle of Cu^2+^ onto MNA was repeated three times. The adsorption capacity of MNA was recycled, and the regeneration of the MNA of the adsorption–desorption cycle of Cu^2+^ was repeated three times using the same MNA. The desorption process was studied for 3 cycles of MNA-7 h. The results proved that the magnetic nanoparticles have higher sustainability for industrial applications. The results also revealed that Cu^2+^ could be desorbed from the adsorbent in the presence of deionized water as the desorbing agent. For repeated use of MNA, the adsorbed Cu^2+^ were desorbed under suitable conditions. In this work, the percentage of desorption by MNA was obtained by 0.1 M HCl as shown in Table 6. Hence, MNA exhibits an enhanced recovery efficiency of 70.87%. Figure 16 displays the desorption efficiency of MNA. The adsorption capacity decreased by 10% during three adsorption–desorption cycles which indicates the stability and reusability of MNA.

**Table 6:** Regeneration and desorption data of copper onto MNA-7 h

	Adsorption			Desorption	
Metal ions	*C*_*o*_ (mg/L)	*q*_*e*_(mg/g)	*% RE*	*q*_*e*_(mg/g)	*% RE*
Cu^2+^	50	4.408	62.61	3.124	70.87

## Conclusions

Novel magnetite nano adsorbent (MNA) from mill scale waste has been successfully synthesized via conventional milling technique and impacted by high-energy ball milling procedure at varying milling time. The high-energy ball milling (HEBM) at 3, 5 and 7 h successfully produced the MNA in the range of 10–25 nm, as confirmed by HRTEM. The HEBM technique was used to reduce the microcrystalline size to nano-sized particles showing the potentials of MNA as an efficient precursor for Cu^2+^ removal in an aqueous solution.

VSM results showing the MNA-7 h possess the highest magnetization property and indicate the best absorbent, with the specific surface area of 5.98 m^2^g^−1^ and the average pore size of 8.01 nm thereby showing the better adsorption capacity. The adsorption of Cu^2+^ on MNA-7 h was confirmed by EDS and FTIR analysis. For the adsorption studies, pH at 5.4, dosage of 0.05 g and 240 min of contact time, the highest adsorption capacity, *q*_e_ and removal efficiency of 4.408 mgg^−1^ and 62.61% were achieved. Also, at the initial concentration of 50 mgL^−1^ Cu^2+^, the *q*_*e*_ of 4.41 mgg^−1^ was recorded. The reusability efficiency of 70.87% was attained even after three cycles of reapplications and desorption. The Temkin adsorption isotherm fits best with a correlation coefficient, *R*^*2*^ of 0.91. Based on these findings, it can be inferred that MNA is a promising precursor for Cu^2+^ removal.

## Data Availability

The datasets generated during and/or analysed during the current study are available from the corresponding author on reasonable request.
